# A novel meta-analysis based on data augmentation and elastic data shared lasso regularization for gene expression

**DOI:** 10.1186/s12859-022-04887-5

**Published:** 2022-08-23

**Authors:** Hai-Hui Huang, Hao Rao, Rui Miao, Yong Liang

**Affiliations:** 1 Provincial Demonstration Software Institute, Shaoguan University, Shaoguan, China; 2 Faculty of Information Technology, Macau University of Science and Technology, Macau, China; 3 The Peng Cheng Laboratory, Shenzhen, China

**Keywords:** Integrative analysis, Meta-analysis, Regularization, Variable selection, Gene expression

## Abstract

**Background:**

Gene expression analysis can provide useful information for analyzing complex biological mechanisms. However, many reported findings are unrepeatable due to small sample sizes relative to a large number of genes and the low signal-to-noise ratios of most gene expression datasets.

**Results:**

Meta-analysis of multi-data sets is an efficient method for tackling the above problem. To improve the performance of meta-analysis, we propose a novel meta-analysis framework. It consists of two parts: (1) *a novel data augmentation strategy*. Various cross-platform normalization methods exist, which can preserve original biological information of gene expression datasets from different angles and add different “perturbations” to the dataset. Using such perturbation, we provide a feasible means for gene expression data augmentation; (2) *elastic data shared lasso (DSL-*${{\varvec{L}}}_{\mathbf{2}}$*).* The DSL-${\mathbf{L}}_{\mathbf{2}}$ method spans the continuum between individual models for each dataset and one model for all datasets. It also overcomes the shortcomings of the data shared lasso method when dealing with highly correlated features. Comprehensive simulation experiment results show that the proposed method has high prediction and gene selection performance. We then apply the proposed method to non-small cell lung cancer (NSCLC) blood gene expression data in order to identify key tumor-related genes. The outcomes of our experiment indicate that the method could be used for identifying a set of robust disease-related gene signatures that may be used for NSCLC early diagnosis or prognosis or even targeting.

**Conclusion:**

We propose a novel and effective meta-analysis method for biological research, extrapolating and integrating information from multiple gene expression datasets.

## Background

The wide application of modern high-throughput biomedical instruments has greatly accelerated the speed of data generation in the field of life sciences. For example, the Gene Expression Omnibus (GEO) of the National Center for Biotechnology Information (NCBI) has collected more than 3.4 million samples. How to accurately screen out the gene markers that are closely related to the diagnosis, treatment, and drug development of complex diseases from the gene expression data is one of the essential problems in genomic research [1–5].

There are three main problems in analyzing gene expression data by using biostatistics and machine learning methods. (1) *Large p and small n*. Gene expression data sets typically contain a large number of genes and a small number of samples [6]. A very few genes are closely related to the target disease, while others are irrelevant. In terms of machine learning, many unrelated genes can introduce noise and may lead to overfitting, further negatively influencing the performance of classifiers [7]; (2) *Batch effect*. Different gene expression data are generated with different processing structures and data platforms, and the expression values are returned with different numerical scales. Such phenomenon is often referred to as the batch effect [8]; (3) *Low reproducibility*. Because the signal-to-noise ratio in many gene expression datasets is usually low, the published gene biomarkers are rarely duplicated in other studies [9].

The meta-analysis of multiple gene datasets to improve the statistical performance of genome research is a promising solution to meet the above challenges [10]. The current gene-expression data meta-analysis can be divided into three types: (1) the first type of method performs analysis based on combining results from different studies. For example, *p* values [11], effect sizes [12, 13], or ranks [14]. These methods tend to gain more power in identifying differentially expressed (DE) genes. But such a method is trivial, and it is easy to lead to false results; (2) the second type of method usually applies a particular cross-platform normalization (CPN) method to remove the batch effect from multiple datasets, subsequently combining multiple datasets into one large data set. Then, the machine learning method can be used to realize the classification and gene selection of the combined dataset. Due to large datasets, such methods often achieve higher result statistical significance than the first type method [15]. However, due to the inherent complexity of biological data, existing CPN methods can only reduce the batch effect of data but can not completely eliminate it. Therefore directly analyzing the integrated data may raise some problems [15, 16]; (3) the third meta-analysis method establishes a unified model on multiple data sets on the basis of no data merging, which is a new research direction of meta-analysis. For example, meta threshold gradient descent regularization [17], meta-lasso [18], meta-nonconvex [19], and data share lasso (DSL) [20]. A discussion of the advantages and disadvantages of the above methods is presented in “Simulation” section. This paper highlights the DSL method because it is more formally concise and reasonable. The DSL method spans the continuum between individual models for each dataset and one model for all datasets. By applying the lasso penalty, the DSL method also achieves gene selection. However, the DSL method does not achieve the grouping effect (strongly correlated genes tend to be included in or omitted from the model altogether [21]) and therefore ignores correlations between the genes and cannot be used to analyze data with dependent structures. If there is a high correlation between a group of genes, the DSL method often only selects one gene representing the whole group. Because genes involved in the same biological pathway are usually highly correlated, group situation is very common in gene expression data [22].

Data augmentation (DA) refers to the appropriate “perturbation” of the original data in order to achieve data set expansion; this is based on certain prior knowledge, and it proceeds on the premise of maintaining specific information [23]. The winning prediction models described in [24–26] all use DA strategies to artificially increase the number of training examples. Previous research aimed at systematically understanding the benefits of increasing data shows that DA can act as a regulator to prevent overfitting and enhance the generalization ability of the model [27]. The validity of DA inspires us to consider applying augmentation to gene expression data. However, traditional DA methods, *e.g.,* rotating or scaling, are inadequate for gene expression data because they are unable to yield sufficient biological explanations.

**Fig. 1:**
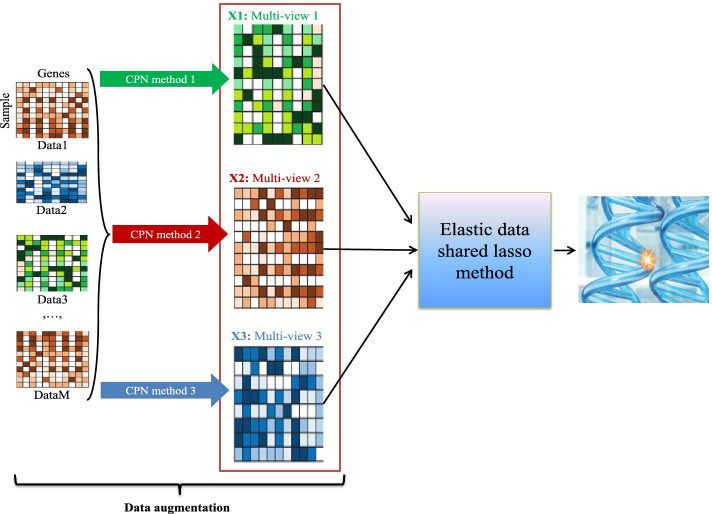
Overview of the proposed DA-DSL-L_2_ meta-analysis framework. Data1, …, DataM are merged by different cross-platform normalization (CPN) methods, respectively, to achieve data augmentation. We process these augmented data (or multi-views) using the elastic shared lasso method that considers both data homogeneity and heterogeneity to obtain better feature selection performance

To improve the power of the meta-analysis, in this paper, we proposed a new meta-analysis framework (DA-DSL-${\mathrm{L}}_{2}$, Fig. 1) based on a new DA strategy and elastic data shared lasso (DSL-${\mathrm{L}}_{2}$) method. It consists of two components: (1) *a novel data augmentation (DA)*. Various CPN methods exist that can preserve original biological information of gene expression datasets from different angles and add different “perturbations” to the dataset. Using such perturbation, we can generate a multi-view representation of the datasets; this is a feasible means of gene expression data augmentation. (2) *DSL-*${L}_{2}$. The DSL-${\mathrm{L}}_{2}$ method overcomes the shortcomings of the DSL method when dealing with the presence of highly correlated features. We apply the DA-DSL-${\mathrm{L}}_{2}$ method to a logistic regression model to fulfill the final model. Then, we perform an analysis of non-small-cell lung cancer (NSCLC) blood-based gene expression data to help identify the gene signatures that can be used for the early diagnosis of NSCLC.

Our experimental result shows that the proposed framework is an effective meta-analysis method, which can identify a group of robust genetic markers related to the disease.

The main contributions of the paper are as follows.

1. This study proposes a novel DA strategy that applies to gene expression data. The new strategy will help increase the size of training samples, increase the value density of biological data, improve the effectiveness of machine learning, and enhance the generalizability of molecular marker research.
2. A novel biomarker selection method DSL-${\mathrm{L}}_{2}$ is proposed. The proposed method improves the performance of DSL methods when dealing with highly correlated data variables. In addition, we discuss the reasons why this method enhances the DSL method theoretically.
3. A refined meta-analysis framework DA-DSL-${\mathrm{L}}_{2}$ for gene-expression value enhancement is proposed. In this framework, data augmentation of gene expression data, the shared biological information (homogeneity) and the unique effect (heterogeneity) across the multi-views (or multi-datasets), and the group effect for the genes are all well-considered.
4. Because the signal-to-noise ratio of blood gene expression is very low, finding useful information in blood data is difficult. We identified 59 genes in NSCLC blood gene expression data by using the proposed method. These 59 gene markers accurately distinguished lung cancer samples from normal samples. The 59 genes were further verified by literature analysis, pathway analysis, gene alteration analysis, survival prediction analysis, and association analysis. The selected genes could be used for peripheral blood testing for the early diagnosis of NSCLC.

The rest of this paper is organized as follows: We reviewed the related works in “Result” section. In “NSCLC data preparation and augmentation” section We describe our data augmentation strategy for the gene expression data, as well as the novel regularization DSL-${\mathrm{L}}_{2}$ technique. In “NSCLC model training and performance” section we present a novel algorithm for the DSL-${\mathrm{L}}_{2}$, and explain how DSL-${\mathrm{L}}_{2}$ strengthening the DSL theoretically. In “Biological analysis for the selected genes from NSCLC” section we measure the performance of our proposed method through a comprehensive simulation analysis and real mRNA expression level data experiment. A brief discussion and conclusion are provided in “Discussion and conclusion” section.

## Result

To test the effectiveness of our proposed method, we conducted a comprehensive simulation test and an evaluation with two large lung cancer gene-expression datasets. The statistical model used here is the logistics regression model.

### Simulation

Four Scenarios are considered in the simulation. Each Scenario consists of three datasets, and each dataset consists of 100 samples with 1000 dimensions. We simulate data from the true model: $y_{k}$ = [Prob ($y_{k}$ = 1|$X_{k}$; $\beta$) > 0.5].

In Scenario 1, we assume that the discrepancy among different datasets is small, which can be expressed as $X_{k} \sim N(\sqrt k - 1,\sqrt k )$, $k{ = }1,2,3.$

The $\beta$ values are simulated from

$$\beta = \left( {\underbrace {{3,3,3,3,3,}}_{5}\underbrace {{0, \ldots ,0}}_{995}} \right),$$

with a grouped variable situation ${x}_{i}=\rho \times {x}_{1}+\left(1-\rho \right)\times {x}_{i}$, i = 2, 3, 4, 5.

Scenario 2 is similar to Scenario 1, except that there are other independent factors also contributing to the corresponding classification variable $\mathrm{y}$:

$$\beta = \left( {\underbrace {{3,3,3,3,3,2, - 2,2,2, - 2,}}_{10}\underbrace {{0, \ldots ,0}}_{990}} \right)$$

In Scenario 3, we consider that the discrepancy among different datasets is significant: $X_{k} \sim N\left( {k - 1,\left( {\frac{k}{\sqrt k }} \right)^{2} } \right)$, $k{ = }1,2,3.$

$$\beta = \left( {\underbrace {{3,3,3,3,3,1.5,2, - 2,2,2, - 2,}}_{10}\underbrace {3, \ldots ,3,}_{20}\underbrace {{0, \ldots ,0}}_{970}} \right)$$

For which we define two grouped variables:

$$\begin{aligned}{x}_{i}&=\rho \times {x}_{1}+\left(1-\rho \right)\times {x}_{i},\quad\mathrm{ i}=2,3,4,5;\\ {x}_{i}&=\rho \times {x}_{11}+\left(1-\rho \right)\times {x}_{i},\quad \mathrm{ i}=12,\dots ,30; \end{aligned}$$

Scenario 4 is similar to Scenario 3, except that we consider a case where there are three grouped variables:

$$\beta = \left( {\begin{array}{*{20}c} {\underbrace {3, \ldots ,3,}_{30}\underbrace {{ - 2.5,2, - 1.5,1.8, - 2.5,}}_{5}\underbrace {{3, \ldots ,3,}}_{40}\underbrace {{2, \ldots ,2,}}_{25}} \\ {\underbrace {3, \ldots ,3,}_{30}\underbrace {2, \ldots ,2,}_{70}\underbrace {{0, \ldots ,0}}_{800}} \\ \end{array} } \right)$$

The three grouped variables are defined as follows:

$$\begin{aligned} {x}_{i}&=\rho \times {x}_{1}+\left(1-\rho \right)\times {x}_{i},\quad\mathrm{ i}=2,\dots ,30;\\ {x}_{i}&=\rho \times {x}_{36}+\left(1-\rho \right)\times {x}_{i},\quad\mathrm{ i}=37,\dots ,75; \\ {x}_{i}&=\rho \times {x}_{101}+\left(1-\rho \right)\times {x}_{i},\quad\mathrm{ i}=102,\dots ,130;\end{aligned}$$

In this case, there are three groups of correlated genes, and some non-correlated genes. A well-sparse regression approach identifies only the 200 true genes, while setting the coefficients of the other 800 noise genes to zero.

We use tenfold cross-validation (CV) on a multi-dimensions procedure and apply it to the training data in order to select the optimal tuning parameter(s) (which balances the tradeoff between data fit and model complexity). In a tenfold CV, the data is firstly divided into 10 equally (or nearly equally) sized folds (or segments); then, 10 iterations of training and validation are conducted. A different fold of the data is held out for validation in every iteration, while the remaining ninefolds are used for model-building. There are three parameters: ${\lambda }_{1}$, ${\lambda }_{2}$, and ${r}_{d}$. The ${r}_{d}$ parameter is set to $\frac{1}{\sqrt{D}}$ as recommended by [20]. The (${\lambda }_{1}$, ${\lambda }_{2}$) gird that maximizes the cross-validation accuracy performance are chosen as the optimal parameters. The ${\lambda }_{1}$ sequence was generated for the X and Y values in such a way that the largest ${\lambda }_{1}$ value is just sufficient to produce all zero coefficients $\beta$. ${\lambda }_{2}$ was chose from {0.001:0.01:5} (Start:Step-Size:End). Lasso and Elastic net were performed using the “glmnet” function (Matlab, version 2014b). The other methods were performed using our own Matlab codes. This simulated data experiment and the following real data experiments are calculated based on a personal computer with Ryzen 7 2700X and 64G RAM.

We set the correlation control variable $\rho$ of genes to 0.3, 0.6, and 0.9, respectively. We ran the experiment 800 times for every method and reported the average 10-CV classification accuracy.

Gene selection is a crucial part of genomic analysis. In our study, the gene selection ability of each approach is measured using Youden’s index (YI). The YI integrates sensitivity and specificity information under situations that emphasize sensitivity and specificity, yielding a value ranging from 0 to 1. A higher YI implies better gene selection performance.

$${\text{YI}} = {\text{Sensitivity}} + {\text{Specificity}}{-}{1}.$$

where Sensitivity $:=\frac{\mathrm{TP}}{\mathrm{TP}+\mathrm{FN}}$, Specificity := $\frac{\mathrm{TN}}{\mathrm{TN}+\mathrm{FP}}$, True Negative (TN) ${:=\left|\overline{\beta }.*\overline{\widehat{\beta } }\right|}_{0}$, False Positive (FP) ${:=\left|\overline{\beta }.*\widehat{\beta }\right|}_{0}$, False Negative (FN) ${:=\left|\beta .*\overline{\widehat{\beta } }\right|}_{0}$ and True Positive (TP) ${:=\left|\beta .*\widehat{\beta }\right|}_{0}$. The $.*$ is the element-wise product, and ${\left|.\right|}_{0}$ calculates the number of non-zero elements in a vector, $\overline{\beta }$ and $\overline{\widehat{\beta } }$ are the logical “not” operators on the vectors $\beta$ and $\widehat{\beta }$.

To accomplish DA, we use three classic cross-platform normalization methods (Z-score normalization, COMBAT, and XPN). For example, we use Z-score normalization to merge the three datasets ($X_{1}$, $X_{2}$ and $X_{3}$) to produce a view of the raw data. Similarly, COMBAT and XPN are used to generate other data views, respectively.

The competing methods can be divided into three groups: (1) *Without considering homogeneity*. The models training in a single dataset $X_{1}$ or $X_{2}$ or $X_{3}$, respectively, include Lasso, Elastic, HLR (we report the average performance of these models for these three data sets). (2) *Without considering heterogeneity*. The three datasets are merged into a merged dataset [$X_{1}$; $X_{2}$; $X_{3}$] by COMBAT. Three models are directly trained on the merged data, including M-Lasso, M-Elastic, and M-HLR. (3) *Without considering the grouping effect.* Such as Meta-Lasso and DSL. Moreover, one classic integrative analysis method Sparse Group Lasso (SGL) [28] is also involved in the experiment.

Table 1 shows the average classification performance on 10-CV for all the methods for 800 runs. In summary, the DA-DSL-${\mathrm{L}}_{2}$ method has certain advantages over the other methods regarding classification evaluations. For example, in Scenario 1 with $\rho$ = 0.3, the mean accuracy attained with the DA-DSL-${\mathrm{L}}_{2}$ method equals 84.19%, which was the best performance among the methods. In Scenario 3 with $\rho$ = 0.6, the value achieved by the DA-DSL-${\mathrm{L}}_{2}$ method equals 79.22%, which is 19.21%, 4.18%, 16.20%, 5.95%, 15.55%, 7.43%, 4.67%, 4.20%, and 3.87% higher than the mean accuracies of Lasso, M-Lasso, Elastic Net, M-Elastic Net, HLR, M-HLR, SGL, Meta-Lasso and DSL, respectively.

**Table 1:** Classification prediction results of the simulation

S	$\rho$	Lasso (%)	M-Lasso (%)	EN (%)	M-EN (%)	HLR (%)	M-HLR (%)	SGL (%)	Meta-Lasso (%)	DSL (%)	DA-DSL-${\mathrm{L}}_{2}$ (%)
		Accuracy									
1	0.3	72.85	80.30	76.65	80.13	74.32	80.18	82.03	82.17	81.39	**84.19**
	0.6	57.32	74.89	59.47	77.47	56.30	80.41	81.52	80.54	79.88	**80.83**
	0.9	58.18	74.55	58.62	75.10	60.88	76.19	79.50	79.60	82.00	**85.68**
2	0.3	51.73	71.55	55.26	71.53	56.60	70.95	75.79	76.34	75.52	**76.37**
	0.6	54.80	71.71	59.73	72.10	56.94	72.39	76.58	73.19	75.41	**77.56**
	0.9	55.96	71.19	60.30	74.70	53.12	72.67	77.01	75.80	77.03	**78.63**
3	0.3	59.08	69.29	61.22	69.10	59.44	68.48	72.34	70.59	74.77	**75.19**
	0.6	60.01	75.04	63.02	73.27	63.67	71.79	74.55	75.02	75.35	**79.22**
	0.9	67.02	70.06	70.05	69.63	68.86	71.99	74.69	72.68	73.01	**75.70**
4	0.3	81.70	84.00	83.58	83.46	80.22	82.02	85.33	83.51	85.88	**87.58**
	0.6	50.01	71.38	53.99	72.96	66.21	73.34	74.74	72.96	74.62	**76.91**
	0.9	61.07	70.32	65.95	71.27	64.97	74.89	80.83	78.71	79.35	**83.50**

*S denotes the Scenario and $\rho$ is the correlation control variable of data. In bold – the best performance amongst all the methods

Table 2 demonstrates the average capability of the gene section by all the approaches for 800 repetitions. Overall, the DA-DSL-L_2_ method achieves the best gene selection performance in all cases. For example, in Scenario 3 with $\rho$ = 0.3, the DA-DSL-L_2_ achieves the superior gene selection performance, with YI = 84.42%, which is 41.05%, 11.45%, 41.15%, 11.29%, 41.59%, 14.76%, 7.62%, 7.90%, and 5.68% higher than that of Lasso, M-Lasso, Elastic Net, M-Elastic Net, HLR, M-HLR, SGL, Meta-Lasso and DSL, respectively. These outcomes imply that the DA-DSL-L_2_ method is able to identify fewer noise genes and more meaningful markers.

**Table 2:** Gene selection results of the simulation

S	$\rho$	Lasso (%)	M-Lasso (%)	EN (%)	M-EN (%)	HLR (%)	M-HLR (%)	SGL (%)	Meta-Lasso (%)	DSL (%)	DA-DSL-${\mathrm{L}}_{2}$ (%)
		Youden’s index									
1	0.3	60.52	92.63	83.31	95.88	90.18	91.31	94.58	93.27	94.78	**96.79**
	0.6	46.08	87.90	47.51	88.58	42.34	78.99	86.74	85.71	86.71	**92.32**
	0.9	41.02	66.80	49.60	83.26	44.34	86.34	73.84	74.52	72.65	**91.29**
2	0.3	43.56	80.44	45.43	83.02	48.95	84.72	83.51	84.41	83.21	**85.89**
	0.6	42.08	76.38	42.53	80.82	48.25	82.10	82.16	80.38	78.18	**84.15**
	0.9	41.98	66.31	41.88	66.41	40.75	67.70	63.43	65.91	65.09	**69.78**
3	0.3	43.37	72.97	43.27	73.13	42.83	69.66	76.80	76.52	78.74	**84.42**
	0.6	49.46	63.47	56.72	81.46	44.80	80.11	73.59	71.15	72.12	**82.37**
	0.9	38.66	57.47	42.25	75.52	39.08	75.53	63.72	62.60	61.57	**80.08**
4	0.3	46.50	50.39	50.24	51.63	47.31	50.42	59.81	57.50	56.23	**68.74**
	0.6	43.53	70.93	43.48	80.29	41.03	79.17	76.26	77.00	79.76	**86.17**
	0.9	43.58	56.48	45.82	73.03	43.73	75.92	65.62	70.76	72.93	**79.78**

*S denotes the Scenario, and $\rho$ is the correlation control variable of data. In bold – the best performance amongst all the methods

The convergence of these methods is also measured. Take Scenario 2 as an example, with a correlation of 0.6 and platform MATLAB or R, it takes 0.08, 0.09, 0.07, 0.07, 4.98, 13.31, 12.29, 9.7, 0.1895, and 3.22 s for the Lasso, M-Lasso,Elastic net, M-Elastic net, HLR, M-HLR, SGL, Meta-Lasso, DSL and DA-DSL-L_2_ to converge to their solutions, respectively.

### Blood-based gene expression signatures in non–small cell lung cancer

Lung cancer remains the main cause of cancer-related deaths around the world. Global average prognosis remains poor, with a 5-year survival rate of about 15% due to late diagnosis of cancer in incorrigible stages in the majority of patients, a source of frustration in therapeutic regimens for advanced disease. It is urgent to establish a more reliable tool for the detection of Non–Small Cell Lung Cancer (NSCLC) in the early stages of the disease before the onset of symptoms.

### NSCLC data preparation and augmentation

To our best knowledge, there are two large (sample size > 150) peripheral whole blood NSCLC gene expression datasets (Table 3). These two datasets are generated from two different platforms, which means that both homogeneity and heterogeneity exist. In this section, we use the proposed method to identify the blood-based gene expression characteristics that can be used for the early diagnosis of NSCLC.

**Table 3:** Briefing of the NSCLC datasets

Datasets [GEO]	Platforms	NSCLC	Controls	Samples
GSE12771 [29]	GPL6102	110	132	242
GSE20189 [30]	GPL571	81	81	162
Total		191	213	404

Each probe set was mapped to an official gene symbol, and for multiple probe sets corresponding to the same gene, we averaged these probe sets to represent the gene. We took the subset of genes common to all datasets. In all, 11,959 genes were reserved.

We randomly select two-thirds of the samples in GSE12771 and GSE20189 for model training. The remaining third of samples in GSE12771 and GSE20189 are used for model testing runs “test set-1” and “test set-2”.

We use three classic cross-platform normalization methods (Z-score normalization, COMBAT, and XPN) to accomplish data augmentation. For example, we use Z-score normalization to merge two training sets (two-thirds of samples in GSE12771 and GSE20189) to produce a view of the raw data. Similarly, COMBAT and XPN are used to generate other data views, respectively. The final training set consists of 846 samples, including 435 NSCLCs and 411 healthy controls; test set-1 consists of 73 samples, including 31 NSCLCs and 42 healthy controls; test set-2 consists of 49 samples, including 23 NSCLCs and 26 healthy controls.

### NSCLC model training and performance

Six strategies are compared with our proposed method: Lasso, Elastic net, HLR, SGL, Meta-Lasso, and DSL.

The tuning regularization parameters of the DA-DSL-${\mathrm{L}}_{2}$ were tuned by using 5-CV on multi-dimensions in the training dataset. A final classifier has been trained with the determining tuning parameters by using all the training data. The model’s cut-off point is calculated as the point that yields the highest YI value.

As shown in Table 4, the DA-DSL-${\mathrm{L}}_{2}$ method outperforms all the competitors in terms of training accuracy, with a training error of only 2.23%. By comparison, lasso achieved a 5.09% training error, almost 2.3 times higher than that of our proposed method. Moreover, the DA-DSL-${\mathrm{L}}_{2}$ method also better than the DSL method (a method that does without achieving grouping effect), which implies that the ${\mathrm{L}}_{2}$ norm technique functions well in gene expression data. The same observation can be seen in the test set-1 and test set-2 results, showing that the proposed method achieves the best classification performance and better efficiency. The test scores predicted by DA-DSL-${\mathrm{L}}_{2}$ method to be an NSCLC patient for cases compared with healthy controls is significant (*P* < 0.01, *t*-test).

**Table 4:** Discrimination results from all methods

Method	Training error	Test-1 accuracy (%)	Test-2 accuracy (%)	No. of selected genes	Convergence time (s)
Lasso	5.09% (0.002)	89.35	84.12	176	11.09
Elastic Net	4.68% (0.001)	91.35	90.12	337	9.52
HLR	4.57% (0.005)	89.98	86.04	139	120.02
SGL	4.42% (0.002)	88.21	85.24	127	63.87
Meta-Lasso	3.68% (0.001)	91.21	90.24	53	70.12
DSL	3.55% (0.000)	92.72	90.88	46	33.87
DA-DSL-L_2_	2.23% (0.002)	94.98	92.16	59	428.65

Standard deviation is shown in brackets

Although the DA-DSL-${\mathrm{L}}_{2}$ method takes a long time among all the techniques. It is superior in terms of feature selection and classification quality.

### Biological analysis for the selected genes from NSCLC 

We provide results for the 10 highest-ranked genes identified by all the strategies in Table 5. As explained in the simulation section above, the proposed method exhibits good performance in identifying the critical gene. Thus, we can argue that the genes identified by the DA-DSL-${\mathrm{L}}_{2}$ method in cancer datasets can help medics attending to cancer patients to deduce the true biomarkers associated with cancer development. For example, EGR1 is linked to cancer suppression due to cell cycle arrest and apoptosis by regulation of cancer suppressor pathways. Patients with low EGR1 expression may be at high risk of disease recurrence, and may have tumors that are resistant to therapy [31]. A recent study reported CD74 gene fusions in patients with lung cancer harboring the kinase domain of the NTRK1 gene that encodes the TRKA receptor. CD74-NTRK1 fusions result in constitutive TRKA kinase development and are oncogenic. Therapy of cells expressing NTRK1 fusions with inhibitors of TRKA kinase activity can restrain autophosphorylation of TRKA and cell growth [32]. The imbalance between VWF secretion and ADAMTS-13 may play an important role in the hypercoagulability form in advanced NSCLC. Nevertheless, the plasma VWF/ADAMTS-13 ratio elevation may serve as the key predictive factor behind mortality in patients with advanced NSCLC [33]. In addition, SIAH proteins play a critical role in many important biological processes. For example, SIAH2 protein expression is significantly enhanced in human lung cancer and may serve as a novel target for lung cancer therapy [34].

**Table 5:** The highest-ranked selected 10 genes were found by the sparse logistic regression methods from the lung cancer dataset

Rank	Lasso	Elastic Net	HLR	SGL	Meta-Lasso	DSL	DA-DSL-${\mathrm{L}}_{2}$
1	PRKAR2B	PVALB	HTRA1	BFSP1	PLSCR1	ATIC	ACAP2
2	PMM2	AMDHD2	C6orf47	PMM2	DSC2	BANK1	NTRK1
3	ARHGAP10	SLC11A2	CD177	P2RY10	TOR4A	CD96	SIAH2
4	PKM	HTRA1	COL17A1	ARHGAP10	P2RY10	NTRK1	VWF
5	PRLR	PKM	ARL17A	PKM	EPB41L2	HP	ECHDC3
6	INA	ELOVL4	IL3	CDR1	NTRK1	ECHDC3	EGR1
7	CYP51A1	CDR1	CNTNAP2	PRLR	PRKAB2	RNASE2	VTI1B
8	NLRP2	HP	VIP	GPALPP1	PRLR	S100A12	CD74
9	CDR1	NLRP2	SERPINA7	NLRP2	GPALPP1	CD74	MAPK4
10	P2RY10	P2RY10	C2orf54	CYP51A1	CD74	VPREB3	METTL9

**Fig. 2:**
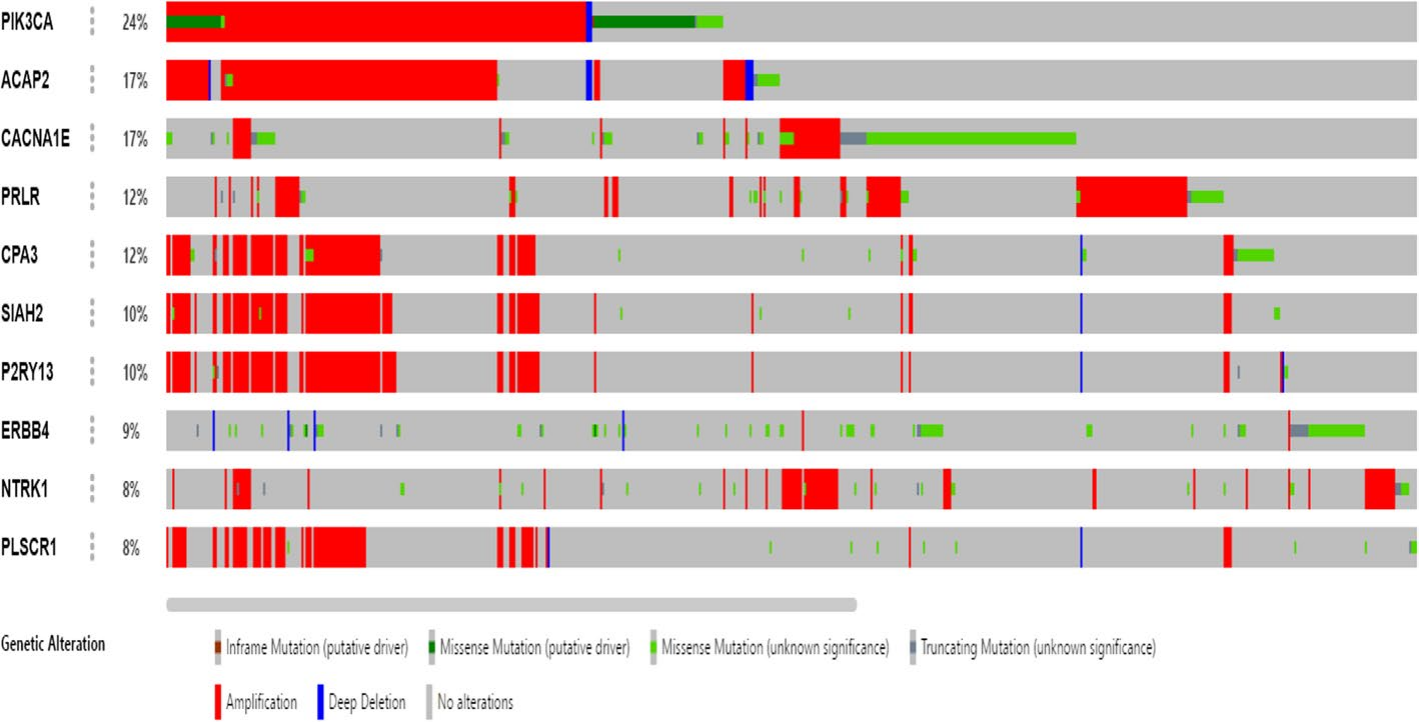
The 10 highest-ranked gene alterations in the TCGA NSCLC cancer (provisional) dataset selected by DA-DSL-${\mathrm{L}}_{2}$

To further validate the selected genes using the proposed method, we perform alterations and pathways analysis using cBioPortal [31] with the NSCLC TCGA dataset and Reactome [35]. It was found that 59 genes were altered in 865 out of 1229 patients with NCSLC (70%). The results of the 10 highest-ranked gene alterations are illustrated in Fig. 2: it can be seen that the maximum alteration gene is PIK3CA (24% alteration in all patients). ACAP2 and CANNA1R alterations were detected in 17% of patients. These results alone provide promising evidence of the therapeutic value of these genes.

**Fig. 3:**
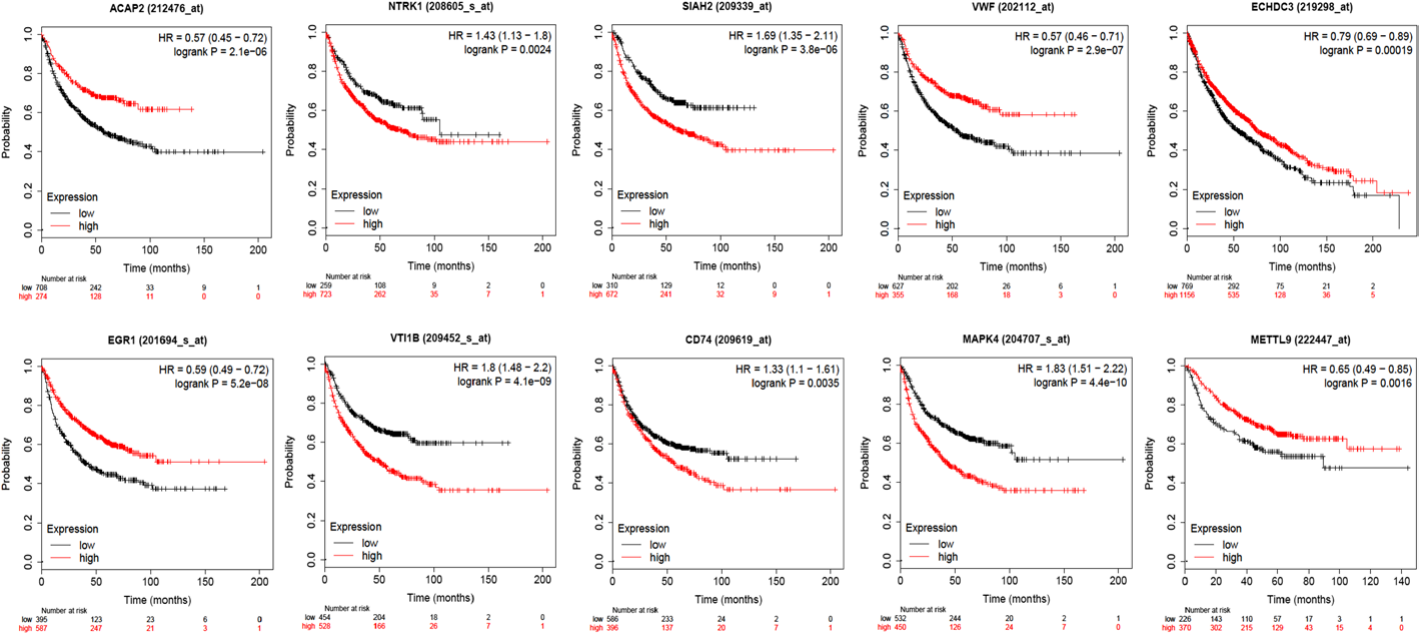
Survival prediction for the top 10 highest-ranked genes selected by DA-DSL-${\mathrm{L}}_{2}$

**Fig. 4:**
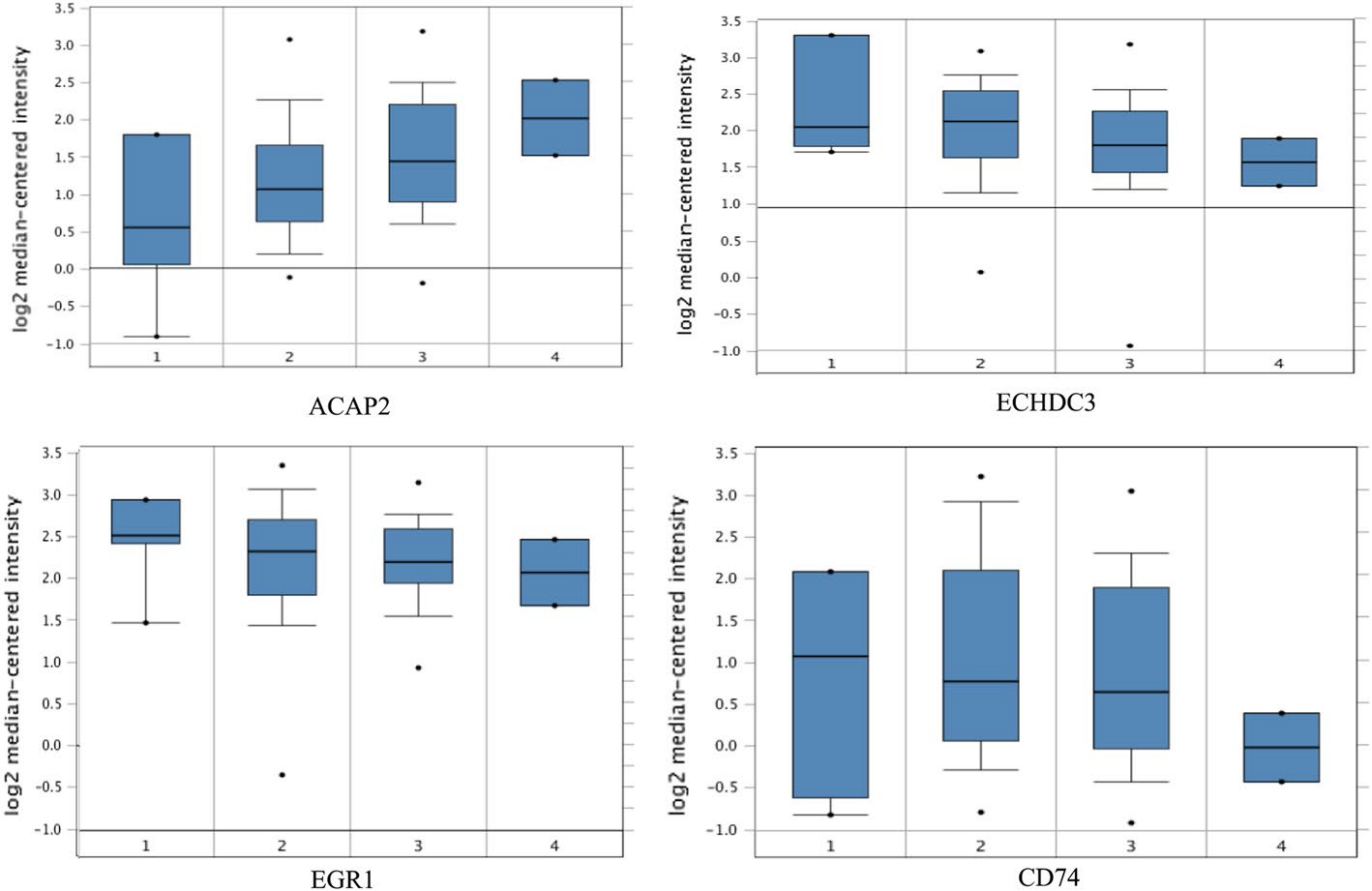
Association between the mRNA expression of ACAP2, ECHDC3, EGR1, and CD74 and tumor grade (grades 1–4) in the Bittner Lung dataset

We also performed a Kaplan–Meier survival analysis (with the help of the *kmplot* web resource) for the 10 genes identified by DA-DSL-${\mathrm{L}}_{2}$ on a union dataset for 1925 patients, the results of which are presented in Fig. 3. Overall, all the genes show a certain prognostic value, for example, MAPK4 (hazard ratio, 1.83; *P* = 4.4e−10), VT11B (hazard ratio, 1.8; *P* = 4.1e−09) and, NTRK1 (hazard ratio, 1.43; *P* = 0.0024). We further validated the 10 genes on the Bittner Lung dataset by Oncomine. As shown in Fig. 4, higher mRNA levels of ACAP2, ECHDC3, EGR1, and CD74 were highly associated with tumor development.

**Fig. 5:**
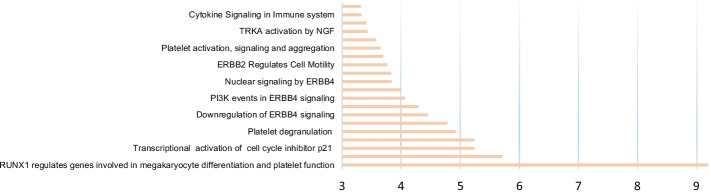
The pathways analysis. Ratio enrichment indicates the functional significance of a gene module with − log(*p* value)

We then performed a pathway analysis for the genes identified using DA-DSL-${\mathrm{L}}_{2}$. Fifty-nine biomarkers are enriched in 153 distinct (with *P* < 0.05) pathways. We summarize the top 20 most significant pathways in Fig. 5.

Of these significant pathways, some of them are platelet function-related. For example, RUNX1 regulates genes involved in megakaryocyte differentiation and platelet function, platelet degranulation, responses to elevated platelet cytosolic Ca2+, and in platelet activation, signaling, and aggregation. It is known that platelets contribute to tumor development via different mechanisms. Metastasis is the major cause of cancer-related death; however, metastasis is a highly inefficient process. Once they enter the bloodstream, cancer cells come into the vicinity of circulating cells and rapidly bind to platelets [36]. Moreover, platelets may help hide cancer from the immune system by inhibiting the function of T cells [37]. Therefore, blood platelets act as local and systemic responders during tumorigenesis and cancer metastasis, and could therefore serve as useful signature sources for the non-invasive detection of cancer [38]. One of the most significant pathways is the Immune-related pathway of Cytokine Signaling. The microenvironment of the primary tumor site mainly includes tumor-associated macrophages, tumor-associated fibroblasts, myeloid-derived suppressor cells, mast cells, etc. These cells secrete various cytokines and chemokines to promote tumor metastasis [39].

Combining the results from Figs. 2, 3, 4 and 5, the gene signatures selected by DA-DSL-${\mathrm{L}}_{2}$ provide potential therapeutic markers and pathways in NSCLC.

### Colorectal cancer study

Colorectal cancer (CRC) is one of the most common neoplastic diseases worldwide. With a high recurrence rate among all cancers, treatment of CRC only improved a little over the last two decades. Early diagnosis and prompt treatment can significantly reduce mortality and morbidity rates. Here data from three gene expression studies are collected and analyzed (Table 6).

**Table 6:** Briefing of the colorectal datasets

Datasets [GEO]	Platforms	Colorectal	Controls	Samples
GSE110223 [40]	GPL96	13	13	26
GSE110224 [40]	GPL15207	14	14	28
GSE113513	GPL570	17	17	34
Total		44	44	88

We primarily follow the data process in the NSCLC study section, such as (1) we took the subset of genes common to all datasets; (2) GSE110223 and GSE110224 are used for model training, and GSE113513 is used for model validation; (3) data augmentation by the three cross-platform normalization methods.

As shown in Table 7, the DA-DSL-${\mathrm{L}}_{2}$ method outperforms all the competitors in terms of training accuracy, with a training error of only 1.15%. The same observation can be seen in the validation result, showing that the proposed method achieves the best classification performance and better efficiency.

**Table 7:** Discrimination results from all methods

Method	Training error	Validation accuracy (%)	No. of selected genes	Convergence time (s)
Lasso	2.41% (0.001)	94.03	33	2.99
Elastic Net	2.10% (0.002)	95.48	65	5.67
HLR	1.88% (0.002)	93.31	52	63.80
SGL	1.62% (0.001)	96.70	67	56.71
Meta-Lasso	2.13% (0.001)	93.63	50	72.05
DSL	2.01% (0.000)	95.82	46	17.50
DA-DSL-L_2_	1.15% (0.000)	98.39	51	43.88

The standard deviation is shown in brackets

With the DA-DSL-L2, fifty-one genes, including CCNA2, DLGAP5, RRM2, are identified in the CRC dataset. These selected genes may play an important role in CRC development. For example, the knockdown of CCNA2 could significantly suppress CRC cell growth by impairing cell cycle progression and inducing cell apoptosis [41]. Some articles showed that CCNA2 is a vital sign to judge the poor prognosis of the tumor, as it is also highly expressed in pancreatic cancer, breast cancer, lung cancer, and other tumors [42]. Clinical studies have shown that DLGAP5 was related to the invasion and migration of CRC [43]. The authors also suggested it is an important measure of poor prognosis. When it comes to the expression of RRM2, studies showed that it is related to the depth of invasion, degree of differentiation, disease-free survival, and metastasis of CRC [44].

**Fig. 6:**
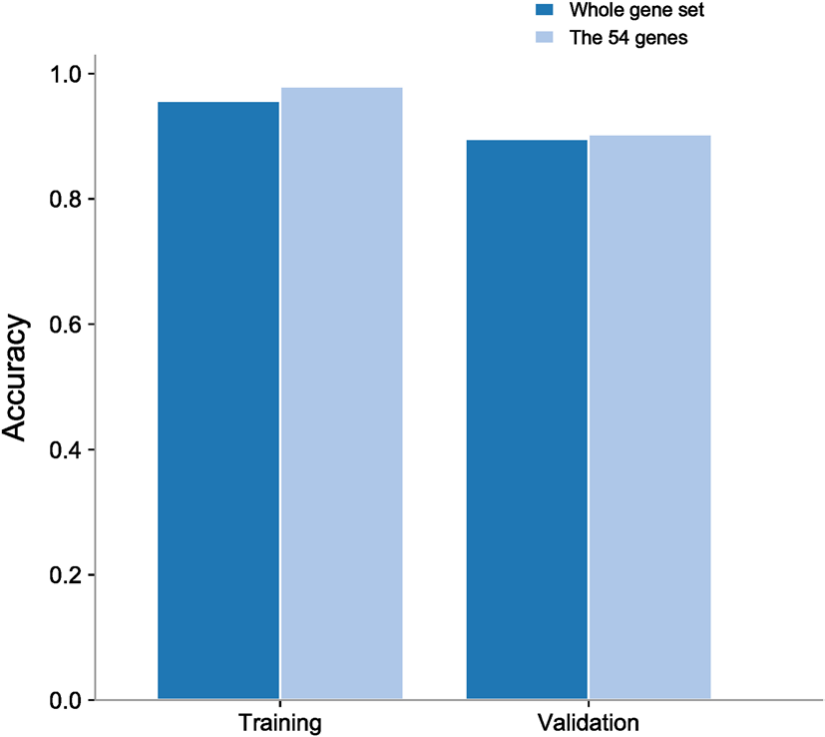
Decision tree performance comparison between 51 genes selected by DA-DSL- L_2_ and a whole gene set consisting of 12,394 genes

To further validate the gene selected by DA-DSL-L_2_, we consider whether the performance improves if a nonlinear classifier such as decision trees is applied to the selected genes (Fig. 6). The result shows promise. The performances of 51 genes are better or equivalent to that of the whole gene set consisting of 12,394 genes.

## Discussion and conclusion

Identifying critical disease-related gene biomarkers is one of the greatest challenges in genomics research. Due to cost considerations, most gene expression data sets in genomic research entail small *n* and large *p*, and there are problems with generalizing conclusions based on these data. Combining multiple experimental data sets in a meta-analysis is one effective way of solving this problem. This research has suggested a novel meta-analysis framework (DA-DSL-L_2_). In this framework, data augmentation of gene expression data, the shared biological information (homogeneity) and the unique effect (heterogeneity) across the multi-views (or multi-datasets), and the group effect for the genes are all well considered.

We have demonstrated a comprehensive simulated experiment. The simulation results of our proposed framework are promising in terms of prediction and gene selection. We have applied the proposed method to NSCLC blood gene expression data to identify key tumor-related genes. Finding knowledge in blood data is challenging because the signal-to-noise ratio in blood gene expression is very low. We generated a multi-view representation based on two large blood-based NSCLC datasets to increase the signal-to-noise ratio. The training sample size ranged up to *n* = 846. We used DSL-${\mathrm{L}}_{2}$ method to process the data. Our results show that the proposed method achieves a superior classification performance (with only 59 gene signatures) compared with six state-of-the-art methods. Moreover, some of the 59 genes are highly coherent across an independent TCGA dataset. Nevertheless, the 59 genes were enriched over 150 significant pathways, some of which are strongly connected with tumor development. We also validated the proposed method on the CRC datasets. The results show that the suggested method outperforms all the competitors in training accuracy. The same observation can be seen in an external CRC validation dataset. In short, we offer a novel and effective meta-analysis strategy for gene expression study that helps turn raw data from multiple gene-expression datasets into knowledge for cancer diagnostics, prognostic and personalized treatment.

Although we focus on the meta-analysis of gene expression data in this paper, the proposed method can be useful for other data types. For example, the proposed data augmentation (DA) strategy in the framework provides a new idea for DA of other non-image data. The proposed feature selection approach (DSL-L_2_) can be directly applied to other data types.

We recommend merging three methods—Z-score, COMBAT, and XPN—for DA. However, this combination might not always be necessary. Some novel merge methods can also be considered, such as scBatch [8]. A more comprehensive examination of the combination with other merge methods will be studied in future research. Other techniques can handle the grouping effect, *i.e.*, the network penalty [45–49]. Future directions may also include incorporating an external gene regulatory network to deal with the grouping effect. As described in the algorithm section, we transform the DSL-${\mathrm{L}}_{2}$ method to a standard Lasso problem. Even though the Lasso problem can be solved by some very efficient method, *i.e*., *glmnet*, to solve a big matrix such as a matrix size of over 40,000 * 40,000 in this paper, this is still a computationally heavy and memory expensive procedure. We, therefore, aim to develop a more efficient algorithm for the DA-DSL-${\mathrm{L}}_{2}$ method in future research. Another weakness of this study is the lack of detailed analysis of the identified genes or pathways.

## Methods

### Meta-analysis

The analysis of data on high-dimensional gene expression is a useful tool for analyzing complex biological mechanisms [3, 4]. However, many reported results are not reproducible or generalized because of the small sample size for a large number of genes and because the signal-to-noise ratio in many gene expression datasets is usually low [9, 50].

There are many publicly available large gene expression studies concerning meta-analysis, a method of combining multiple datasets or other relevant information to improve the statistical power. The current gene-expression data meta-analysis can be divided into three groups: the first group of the method is to perform analysis based on combining results from different studies. For example, For example, *p* value [11], effect size [13], rank [14], adaptively Fisher’s method [51]. For an extensive review of these methods, see [15]. However, such methods ignore the correlations between genes. Hughey and Butte [52] proposed a meta-analysis method to resolve this problem based on the elastic net technique. In Hughey and Butte study, a CPN method is required to remove the batch effect amongst the mutil-datasets. However, due to the inherent complexity of biological data, existing CPN methods can only reduce but not completely eliminate the batch effect of data. Thus, directly analyzing the integrated data may cause issues [16]. Without the procedure of CPN, Ma et al. [17] proposed a meta threshold gradient descent regularization method. By considering the joint modeling of multiple genes, the proposed method can account for the joint effects of genes on clinical outcomes. However, such a method performs gene selection in an “all-in-or-all-out” scene; that is, the method considers the important or unimportant genes in all datasets. Data heterogeneity in meta-analyzed data is common, due to the different experiment conditions, process flows, choices of biospecimens, and platforms. Therefore, if a gene is important in one dataset, it may be unimportant in other datasets.

Li et al. [18] proposed the Meta-lasso method to account for data heterogeneity. Through hierarchical decomposition into regression coefficients, this method can not only lend the power of multiple data sets to increase the power of identifying important genes, but also maintain the flexibility of choice between data sets to consider the heterogeneity of data sets. With a similar idea, Zhang et al. [19] proposed Meta-nonconvex to perform meta-analysis based on nonconvex penalties such as SCAD and MCP. However, Meta-lasso or Meta-nonconvex suffers an “all-out” scene; ignoring variables may be significant on some data sets. Gross [20] proposed the DSL technique. This method spans the continuum between individual models for each dataset and one model for all datasets. By applying the lasso penalty, the DSL method also achieves gene selection. However, the DSL method fails to produce a grouping effect and therefore ignores correlations between genes. When dealing with data that contain group structure and when the genes within the group are highly correlated, the DSL method can only select one gene to represent the entire group structure. In genetic research, genes usually co-express biological functions in the form of pathways (or groups). Some works were suggested to resolve the issue of the highly correlated genes. For example, the elastic net [21], an integration of lasso and ridge (or L_2_ penalty) method, by the L_2_ penalty in the model, grouping effect can be achieved. Based on the same idea, scholars have successively proposed Elastic SCAD [53], SCAD-${\mathrm{L}}_{2}$ [54] and HLR [55, 56].

### Data augmentation

DA is widely applied by computer vision researchers. Models trained through DA are generally more robust and less overfitting [57, 58]. DA requires appropriate “perturbation” of the original data in order to achieve data set expansion; this is based on certain prior knowledge, and it proceeds on the premise of maintaining specific information [23]. The effectiveness of DA has inspired us to consider applying the data augmentation technique to gene expression data. However, traditional DA methods, *e.g.,* rotating or scaling, are not suitable for gene expression data as they do not yield sufficient biological explanations.

CPN is an important procedure for some gene expression meta-analyses. Such an approach removes differences (or batch effect) between different gene expression datasets while preserving biological information within the data. There are extensive efforts in CPN method development. For example, Z-score normalization [59], is perhaps the simplest way of achieving CPN. More advanced methods have been devised, including Distance-weighted discrimination (DWD) [60]. Each source subset is shifted in the DWD direction, by an appropriate amount, through the subtraction of the DWD direction vector multiplied by each projected mean for each gene. Empirical Bayes (or COMBAT) [61] is a Bayes empirical framework for “borrowing information” across genes and experimental conditions, in the hope that the borrowed information will lead to better estimates or more stable conclusions. XPN [62] is a technique involving search blocks of the gene in multiple datasets with non-heterogeneous genes. PLIDA [63], a method that uses topic models to combine the expression patterns in every dataset before standardizing the topics learned with each data set using per-gene multiplication weights. The WaveICA [64] method uses the time trend of the samples in order of injection, breaks down the original data into multi-scale data with different features, extracts and eliminates the effect batch on the multi-scale data, and obtains clean data. Each CPN method preserves the original biological information of the original dataset from different angles and adds different “perturbations” to the dataset. Using such perturbation, we can generate a multi-view representation of the dataset; this is feasible for gene expression data augmentation.

### Data augmentation of the gene expression data

When performing integrative analysis for multiple-gene expression datasets, the batch effect amongst the data usually needs to be eliminated. There exist several proposed methods for removing the batch effect, including DWD [60], disTran [65], Median Rank Score (MRS) [66], Empirical Bayes (ComBat) [61], XPN [62], PLIDA [63], and WaveICA [64]. These CPN methods involve merging data from different aspects and generating different system perturbations. The idea of perturbations is pivotal to DA. In this paper, we propose merging datasets via different merging methods to generate multiple views of the original data. In other words, the gene expression data are augmented via different CPN methods. For example, if there are two datasets with 20 and 30 samples, respectively. We can generate three views of the original data by three CPN methods. The data volume will increase from 50 to 50 * 3 = 150.

### Elastic data shared lasso regularization

The original purpose of the DSL was to address problems arising from observations belonging to non-overlapping, pre-specified groups. In this paper, we extend the DSL method to meta-analysis. More formally, we assume we have *n* observations of the form $({\varvec{x}}_{{\varvec{i}}} ,y_{i} ,d_{i} )$, whereby ${\varvec{x}}_{{\varvec{i}}} \in {\mathbf{\mathbb{R}}}^{p}$, $y_{i} \in {\mathbf{\mathbb{R}}}$, and $d_{i} \in \{ 1,2,...,D\} .$ Here, *p* denotes the number of genes and *D* corresponds to the number of datasets (or views). We define ***X*** as the matrix that has the ${\varvec{x}}_{{\varvec{i}}}$’s as rows, ${\varvec{y}} = (y_{1} ,y_{2} ,...,y_{n} )$, and ${\varvec{d}} = (d_{1} ,d_{2} ,...,d_{n} )$. Without loss of generality, the predictors and responses are all normalized and centered. For simplicity, we consider a regression case, in which we argue that $y_{i}$ is defined as:

1 $$y_{i} = x_{i}^{T} (\beta + \Delta_{{d_{i} }} ) + \varepsilon_{i} ,$$

whereby the $\varepsilon_{i}$ are independent noise terms. The standard DSL is presented as follows:

2 $$(\hat{\beta },\hat{\Delta }_{1} ,...,\hat{\Delta }_{G} ) = {\text{argmin}}\,\frac{1}{2}\sum\limits_{i} {\left( {y_{i} - x_{i}^{T} (\beta + \Delta_{{d_{i} }} )} \right)^{2} + \lambda \left( {\left\| \beta \right\|_{1} + \sum\limits_{d = 1}^{D} {r_{d} } \left\| {\Delta_{d} } \right\|_{1} } \right)}$$

whereby λ is the tuning parameter, $r_{d}$ is used as the regularization parameter over datasets and controls the amount of sharing between the datasets, *β* represents a common effect that is shared across datasets, and $\Delta_{{d_{i} }}$ represents a unique effect for the *i*th dataset. The common effect here is correspondence to the shared biological information, and the unique effect here is correspondence to the discrepancy among different merging methods. However, the DSL method tends to select only one gene to represent the correlated group; genes that perform a similar function are often correlated. This drawback may lead to deterioration in the performance of the DSL method. To overcome this issue, in this paper, we propose an elastic data shared Lasso (DSL-${\mathrm{L}}_{2}$) method, which is encapsulated in the following equation:

3 $$\begin{aligned} (\hat{\beta },\hat{\Delta }_{1} ,...,\hat{\Delta }_{D} ) &= \arg \min \frac{1}{2}\sum\limits_{i} {\left( {y_{i} - x_{i}^{T} (\beta + \Delta_{{d_{i} }} )} \right)^{2} + \lambda_{1} \left( {\left\| \beta \right\|_{1} + \sum\limits_{d = 1}^{D} {r_{d} } \left\| {\Delta_{d} } \right\|_{1} } \right)}\\& \quad + \lambda_{2} \left( {\left\| \beta \right\| + \sum\limits_{d = 1}^{D} {r_{d} } \left\| {\Delta_{d} } \right\|} \right) \end{aligned}$$

whereby the first part is a linear loss function, and the second part is the lasso method used to produce a sparsity on $\beta$ and $\Delta_{d}$; the last part is the ${\mathrm{L}}_{2}$ method or ridge method, which generates a grouping effect on $\beta$ and $\Delta_{d}$. The lambda ${\mathrm{L}}_{1}$ and lambda ${\mathrm{L}}_{2}$ are tuning parameters that control the sparsity and grouping effect, respectively.

Finally, we combine DA with DSL-${\mathrm{L}}_{2}$ method (DA-DSL-${\mathrm{L}}_{2}$) for meta-analysis.

### Solution

In this section, an efficient method is developed to solve the DSL-${\mathrm{L}}_{2}$ problem. It turns out that solving problem (3) is equivalent to a ${\mathrm{L}}_{1}$-type optimization problem.

#### **Lemma 1**

*We define* Z, W *as*

$$\begin{aligned} Z_{N \times ((D + 1) \times P)} & = \left( {\begin{array}{*{20}c} {X_{1} } & {\frac{1}{{r_{1} }}X_{1} } & 0 & {...0} \\ {X_{2} } & 0 & {\frac{1}{{{{r}}_{2} }}X_{2} } & {...0} \\ \vdots & & & \\ {X_{D} } & 0 & 0 & {...\frac{1}{{{{r}}_{D} }}X_{D} } \\ \end{array} } \right), \\ W_{((D + 1) \times P) \times ((D + 1) \times P)} & = \left( {\begin{array}{*{20}c} {\sqrt {\lambda_{2} } {\mathbf{I}}_{P} } & 0 & 0 & {...0} \\ 0 & {\sqrt {\lambda_{2} } \frac{1}{{\sqrt {r_{1} } }}{\mathbf{I}}_{P} } & 0 & {...0} \\ \vdots & & {\sqrt {\lambda_{2} } \frac{1}{{\sqrt {r_{2} } }}{\mathbf{I}}_{P} } & \vdots \\ 0 & 0 & 0 & {...\sqrt {\lambda_{2} } \frac{1}{{\sqrt {r_{D} } }}{\mathbf{I}}_{P} } \\ \end{array} } \right), \\ \end{aligned}$$

*where* $X_{k}$ and $y_{k}$ *represent the dataset* *k* (*or view* *k*). *We also define* $$X^{*} { = }(1{ + }\lambda_{2} )^{ - 1/2} \left( {\begin{array}{*{20}c} Z \\ W \\ \end{array} } \right)$$, $\tilde{y} = (y_{1}^{T} ,y_{2}^{T} ,...,y_{D}^{T} )^{T}$, $$\tilde{y}^{*} = \left( {\begin{array}{*{20}c} {\tilde{y}} \\ 0 \\ \end{array} } \right)$$, $\tilde{\beta } = \sqrt {1 + \lambda_{2} } (\beta^{T} ,r_{1} \Delta_{1}^{T} ,...,r_{D} \Delta_{D}^{T} )^{T}$, $\tilde{\beta }^{*} = \sqrt {1 + \lambda_{2} } \tilde{\beta }$ *and* $\gamma { = }\lambda_{1} /\sqrt {1 + \lambda_{2} }$.

*Then we have*

4 $$\begin{aligned} \frac{1}{2}\left\| {\tilde{y}^{*} - X^{*} \tilde{\beta }^{*} } \right\|^{2} + \gamma \left\| {\tilde{\beta }^{*} } \right\|_{1} &= \frac{1}{2}\sum\limits_{i} {\left( {y_{i} - x_{i}^{T} (\beta + \Delta_{{d_{i} }} )} \right)^{2} + \lambda_{1} \left( {\left\| \beta \right\|_{1} + \sum\limits_{d = 1}^{D} {r_{d} } \left\| {\Delta_{d} } \right\|_{1} } \right)} \\ &\quad+ \lambda_{2} \left( {\left\| \beta \right\| + \sum\limits_{d = 1}^{D} {r_{d} } \left\| {\Delta_{d} } \right\|} \right) \end{aligned}$$

*Let* $\beta^{*}$ *be the solver to the above lasso problem*, *i.e.*,

5 $$\hat{\beta }^{*} = \mathop {\arg \min }\limits_{{\tilde{\beta }^{*} }} \frac{1}{2}\left\| {\tilde{y}^{*} - X^{*} \tilde{\beta }^{*} } \right\|^{2} + \gamma \left\| {\tilde{\beta }^{*} } \right\|_{1} ,$$

*then the solution to* Eq. (3) *becomes*

$$\hat{\beta } = \frac{1}{{\sqrt {1 + \lambda_{2} } }}\hat{\beta }^{*} .$$

The proof is just simple algebra, which we omit. Lemma 1 shows that the DSL-${\mathrm{L}}_{2}$ can perform an automatic gene selection in a way similar to the lasso, and can be solved by many efficient methods, such as the Matlab/R package “glmnet” [67]. A type algorithm to solve lasso is the coordinate descent algorithm (CDA). The algorithm is widely applied for solving optimization models, especially for small n and big p problems. This is because the complexity of the asymptotic time of CDA is just O(*npm*), where *n*,* p* and* m* represent the numbers of training sample size, iteration, and features, respectively. Typically, *n* and *m* are not large.

We now prove the DSL-${\mathrm{L}}_{2}$ method improves the prediction power of DSL to some extent.

#### Theorem 1

*With the* *Lemma* 1, *the DSL*-${\mathrm{L}}_{2}$ *estimates* $\widehat{\beta }$ *are given by*

6 $$\overset{\lower0.5em\hbox{$\smash{\scriptscriptstyle\frown}$}}{\beta } = \mathop {\arg \min }\limits_{{\tilde{\beta }^{*} }} \tilde{\beta }^{*{\text{T}}} \left( {\frac{{Z^{{\text{T}}} Z + \lambda_{2} u{\mathbf{I}}}}{{1 + \lambda_{2} }}} \right)\tilde{\beta }^{*} - 2\tilde{y}^{{\text{T}}} Z^{{\text{T}}} \tilde{\beta }^{*} + \lambda_{1} \left| {\tilde{\beta }^{*} } \right|_{1} .$$

*where* $u = 1 + \frac{1}{{r_{1} }} + \frac{1}{{r_{2} }} + \cdots + \frac{1}{{r_{D} }}$. *The DSL regularization can be rewritten as*

7 $$\overset{\lower0.5em\hbox{$\smash{\scriptscriptstyle\frown}$}}{\beta } ({\text{DSL}}) = \mathop {\arg \min }\limits_{{\tilde{\beta }^{*} }} \tilde{\beta }^{{\text{*T}}} Z^{{\text{T}}} Z\tilde{\beta }^{*} - 2\tilde{y}^{{\text{T}}} Z^{{\text{T}}} \tilde{\beta }^{*} + \lambda_{1} \left| {\tilde{\beta }^{*} } \right|_{1} .$$

*Theorem* 1*implies the* DLS-$${\mathrm{L}}_{2}$$ *approach is an improved version of the DSL method. Note that* $$\overset{\lower0.5em\hbox{$\smash{\scriptscriptstyle\frown}$}}{\Sigma } = Z^{{\text{T}}} Z$$ *is a sample version of the correlation matrix* $$\Sigma$$ *and*

$$\frac{{Z^{{\text{T}}} Z + \lambda_{2} u{\mathbf{I}}}}{{1 + \lambda_{2} }} = (1 - \sigma )\overset{\lower0.5em\hbox{$\smash{\scriptscriptstyle\frown}$}}{\Sigma } + \sigma u{\mathbf{I}},$$

*where* $$\sigma = \lambda_{2} /(1 + \lambda_{2} )$$ *shrinks* $$\overset{\lower0.5em\hbox{$\smash{\scriptscriptstyle\frown}$}}{\Sigma }$$ *that towards the identity matrix. The prediction accuracy can often be improved by changing* $$\overset{\lower0.5em\hbox{$\smash{\scriptscriptstyle\frown}$}}{\Sigma }$$ *to a more shrunken estimate in the linear discriminate analysis* [68, 69]. *Thus, the DSL*-$${\mathrm{L}}_{2}$$ *strengthen the DSL method by regularizing* $$\overset{\lower0.5em\hbox{$\smash{\scriptscriptstyle\frown}$}}{\Sigma }$$ in Eq. (6). *The proof of* Theorem 1*is presented in the* “Appendix”.

## Data Availability

All data used in this manuscript are downloaded at GEO (http://www.ncbi.nlm.nih.gov/geo) with access numbers [GSE12771, GSE20189, GSE110223, GSE110224, and GSE113513].

## Appendix: Proof

### *Proof of Theorem 1*

Let $\hat{\beta }$ be the DSL-L2 solution. By definition and Eq. (5), we have.

8 $$\begin{aligned} \hat{\beta } & = \mathop {\arg \min }\limits_{{\tilde{\beta }^{*} }} \left| {\tilde{y}^{*} - {{X}}^{*} \frac{{\tilde{\beta }^{*} }}{{\sqrt {1 + \lambda_{2} } }}} \right|^{2} + \frac{{\lambda_{1} }}{{\sqrt {1 + \lambda_{2} } }}\left| {\frac{{\tilde{\beta }^{*} }}{{\sqrt {1 + \lambda_{2} } }}} \right|_{1} \\ & = \mathop {\arg \min }\limits_{{\tilde{\beta }^{*} }} \tilde{\beta }^{{*\text{T}}} \left( {\frac{{{{X}}^{{{*}{\text{T}}}} {{X}}}}{{1 + \lambda_{2} }}} \right)\tilde{\beta }^{*} - 2\frac{{\tilde{y}^{{*{\text{T}}}} {{X}}^{*} }}{{\sqrt {1 + \lambda_{2} } }} + \tilde{y}^{{*{\text{T}}}} \tilde{y}^{*} + \frac{{\lambda_{1} \left| {\tilde{\beta }^{*} } \right|_{1} }}{{1 + \lambda_{2} }}. \\ \end{aligned}$$

Substituting the identities

$$\begin{aligned} {{X}}^{{*{\text{T}}}} {{X}} & = \frac{{Z^{{\text{T}}} Z + \lambda_{2} u{\mathbf{I}}}}{{1 + \lambda_{2} }}, \\ \tilde{y}^{{*{\text{T}}}} {{X}}^{*} & = \frac{{\tilde{y}^{{\text{T}}} Z}}{{\sqrt {1 +\lambda_{2} } }}, \\ \tilde{y}^{{*{\text{T}}}} \tilde{y}^{*} & = \tilde{y}^{{\text{T}}} \tilde{y} \\ \end{aligned}$$

into Eq. (8), we have

$$\begin{aligned} \overset{\lower0.5em\hbox{$\smash{\scriptscriptstyle\frown}$}}{\beta } & = \mathop {\arg \min }\limits_{{\tilde{\beta }^{*} }} \frac{1}{{1 + \lambda_{2} }}\left\{ {\tilde{\beta }^{{*{\text{T}}}} \left( {\frac{{Z^{{\text{T}}} Z + \lambda_{2} u{\mathbf{I}}}}{{1 + \lambda_{2} }}} \right)\tilde{\beta }^{*} - 2\tilde{y}^{{\text{T}}} Z^{{\text{T}}} \tilde{\beta }^{*} + \lambda_{1} \left| {\tilde{\beta }^{*} } \right|_{1} } \right\} + \tilde{y}^{{\text{T}}} \tilde{y} \\ & = \mathop {\arg \min }\limits_{{\tilde{\beta }^{*} }} \tilde{\beta }^{{\text{*T}}} \left( {\frac{{Z^{{\text{T}}} Z + \lambda_{2} u{\mathbf{I}}}}{{1 + \lambda_{2} }}} \right)\tilde{\beta }^{*} - 2\tilde{y}^{{\text{T}}} Z^{{\text{T}}} \tilde{\beta }^{*} + \lambda_{1} \left| {\tilde{\beta }^{*} } \right|_{1} . \\ \end{aligned}$$

## Abbreviations

GEO: Gene Expression Omnibus
CPN: Cross-platform normalization
DSL: Data share lasso
DA: Data augmentation
DA-DSL-${\mathrm{L}}_{2}$: Elastic data shared lasso with a new DA strategy
DSL-${\mathrm{L}}_{2}$: Elastic data shared lasso
NSCLC: Non-small-cell lung cancer
CRC: Colorectal cancer
DWD: Distance-weighted discrimination
CDA: Coordinate descent algorithm
